# The association between energy‐adjusted dietary inflammatory index and 10‐year cardiovascular risk: Fasa adult cohort study

**DOI:** 10.1002/fsn3.4181

**Published:** 2024-05-13

**Authors:** Matin Sepehrinia, Hossein Pourmontaseri, Mohammad Mehdi Naghizadeh, Farhad Vahid, James R. Hebert, Reza Homayounfar, Abdulhakim Alkamel

**Affiliations:** ^1^ Student Research Committee Fasa University of Medical Sciences Fasa Iran; ^2^ Non‐Communicable Diseases Research Center Fasa University of Medical Sciences Fasa Iran; ^3^ Department of Precision Health, Nutrition and Health Research Group Luxembourg Institute of Health Strassen Luxembourg; ^4^ Department of Epidemiology and Biostatistics, Arnold School of Public Health University of South Carolina Columbia South Carolina USA; ^5^ South Carolina Statewide Cancer Prevention and Control Program University of South Carolina Columbia South Carolina USA; ^6^ National Nutrition and Food Technology Research Institute (WHO Collaborating Center), Faculty of Nutrition Sciences and Food Technology Shahid Beheshti University of Medical Sciences Tehran Iran; ^7^ Department of Cardiovascular Disease, Faculty of Medicine Fasa University of Medical Sciences Fasa Iran

**Keywords:** diet, inflammation, prevention, pro‐inflammatory diet, risk factor, risk prediction

## Abstract

A healthy diet is dominant in cardiovascular disease (CVD) prevention. Inflammation is pivotal for CVD development. This study aimed to evaluate the association between the pro‐inflammatory diet and the CVD risk. This cross‐sectional study involved 10,138 Fasa adult cohort study participants. After excluding participants with missing data, the Energy‐Adjusted Dietary Inflammatory Index (E‐DII) was calculated to assess the inflammatory potential of diet using the recorded Food Frequency Questionnaire. Framingham risk score (FRS) was used to predict the 10‐year risk of CVD. The association between E‐DII and high risk for CVD was investigated using multinominal regression. After exclusion, the mean age of studied individuals (*n* = 10,030) was 48.6 ± 9.6 years, including 4522 men. Most participants were low risk (FRS <10%) for CVD (87.6%), while 2.7% of them were high risk (FRS ≥20%). The median FRS was 2.80 (1.70, 6.30). The E‐DII ranged from −4.22 to 4.49 (mean E‐DII = 0.880 ± 1.127). E‐DII was significantly associated with FRS. This result persisted after adjusting for confounding factors and in both genders. This study revealed that the pro‐inflammatory diet significantly increases the CVD risk. Consequently, reducing the inflammatory potential of diet should be considered an effective dietary intervention in CVD prevention.

## Graphical abstract

Individuals with a higher E‐DII score had a higher risk of cardiovascular diseases. Preventive strategies should focus on reducing the inflammatory potential of diet by encouraging people to consume diets rich in fiber‐containing food and lower amounts of saturated fatty acids and simple carbohydrates.

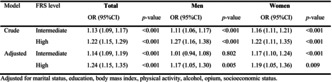

## 1 INTRODUCTION

Although modifiable cardiovascular risk factors have been known and preventive strategies have been taken into action for decades, cardiovascular diseases (CVD) are still the leading cause of mortality globally. About 18.6 million people died because of CVD in 2019 (Roth, Mensah, Johnson, et al., 2020). This number will rise in the future as the population continues to age and the prevalence of cardiovascular risk factors (such as obesity, hypertension, and diabetes) increases (Dahlöf, 2010). Inadequate control of CVD risk factors is the major cause of failure in preventive strategies (Roth, Mensah, & Fuster, 2020). Hence, accurate perception and management of CVD risk factors are pivotal. Using risk prediction models helps to identify high‐risk individuals based on their CVD risk factors to choose the appropriate preventive strategy (Law et al., 2015). Framingham risk score (FRS) is the first and most well‐known risk score developed to predict CVD risk. FRS was designed based on the data from the Framingham study and developed over time, lastly in 2008 (D'Agostino et al., 2008, 2013).

Inflammation is the main part of CVD pathophysiology (Libby, 2021a). Lipid accumulation underneath the tunica intima layer of the arterial wall and endothelial dysfunction trigger the inflammatory process (Alfaddagh et al., 2020). This inflammatory response contributes to both innate and adaptive immunity and is mediated by factors such as smoking, diet, and physical activity. Immune cells such as macrophages and type 1 T‐helper lymphocytes (Th1) in a joint action with inflammatory molecules, namely interleukin‐1 (IL‐1), necrosis factor‐alpha (TNF‐α), and interferon‐gamma (IFN‐γ), foster atherogenesis. However, Th2 and T‐regulatory cells, in collaboration with IL‐10 and transforming growth factor‐beta (TGF‐β), mitigate inflammation and atherosclerosis formation (Alfaddagh et al., 2020; Libby, 2021a, 2021b).

Lifestyle modification, including dietary intervention, smoking cessation, and regular physical activity, has always been a prime part of prevailing guidelines on CVD prevention (Arnett et al., 2019; Visseren et al., 2022). Diet affects systematic inflammation and changes in cytokines and interleukin levels in the serum. For instance, studies rendered that consumption of diets enriched with trans fatty acids (TFA), saturated fatty acids, and high‐glycemic‐index carbohydrates are associated with increased levels of C‐reactive protein (CRP) and inflammation; in contrast, nutrients such as magnesium, fiber, and carotenoid decrease inflammatory markers (Alfaddagh et al., 2020; Galland, 2010; Mazidi et al., 2017; Mozaffarian, 2006). To assess the inflammatory potential of every person's diet, the dietary inflammatory index (DII) was developed based on nutrient intake and changes in inflammatory markers (Cavicchia et al., 2009). The DII is a validated tool for scoring the effect of diet on inflammation and has been linked to several health conditions such as diabetes (King & Xiang, 2019), nonalcoholic fatty liver disease (NAFLD) (Valibeygi et al., 2023), cancers, and all‐cause mortality (Farazi et al., 2021). However, the DII could not estimate the inflammatory potential of nutrients independent of the energy intake. Therefore, the energy‐adjusted dietary inflammatory index (E‐DII) was developed to predict the inflammatory potential of nutrients per 1000 kilocalories (Hébert et al., 2019). Higher E‐DII scores indicate a pro‐inflammatory diet, whereas lower E‐DII scores indicate an anti‐inflammatory diet.

Studies have linked DII to cardiometabolic risk factors and CVD (Ji et al., 2020), but studies evaluating the relationship between the E‐DII score and CVD risk are still scarce. Recently, a cross‐sectional study has evaluated the relationship between E‐DII and FRS (Wang et al., 2021), but the sample size was small, and all participants are female. So, the present study aimed to investigate the association between E‐DII and CVD risk assessed with Framingham risk score (FRS) in a large number of participants from the Fasa Adult Cohort Study (FACS), which includes large numbers of men and women.

## 2 METHODS

### 2.1 Study design

The present cross‐sectional study was conducted on the population of FACS (*n* = 10,138), which included adult participants over 35 years old. The participants with missing data (*n* = 108) were excluded from the studied population. In the first phase of FACS, comprehensive questionnaires, including demographic characteristics, anthropometrics, medical history, and Food Frequency Questionnaire (FFQ), were gathered from individuals from Sheshdeh, Fasa, Iran. All evaluated variables were collected from the database of FACS, which were recorded based on the data collection protocol of FACS (Homayounfar et al., 2023).

### 2.2 Measurements

Demographic features, including age (year), gender (male, female), body mass index (kg/m^2^), ethnicity (Fars, Turkish, Arab, and others), jobs, educational level (illiterate, primary school, secondary school, high school, and university), socioeconomic status (Asset Index), physical activity (metabolic equivalent of tasks, MET), marital status (single, married, widow, or divorced), and disease (CVD, myocardial infarction, stroke, chronic obstructive pulmonary disease, diabetes mellitus, hypertension, chronic kidney disease, nonalcoholic fatty liver disease, thyroid disorders, renal stones, and gallbladder stones), were gathered from database of FACS (Farjam et al., 2016).

Participants' dietary intake was evaluated using a semi‐quantitative FFQ containing 125 food items. The FFQ, adapted from the Willett format questionnaire (Willett et al., 1985), was tailored for Iranian participants to reflect the typical foods consumed in Iran. Each food item was assigned a standard portion size based on the United States Department of Agriculture (USDA) serving sizes (Farjam et al., 2016). The validity and details of the FFQ were explained elsewhere (Eghtesad et al., 2023).

The quantitative variables were categorized to simplify the statistical comparison of demographic features among FRS subgroups. The age, physical activity, and socioeconomic status were categorized into quartiles. Body mass index was defined as the level of obesity (<0.25 kg/m^2^, normal; 25–30 kg/m^2^, overweighted; >30 kg/m^2^, obese). Educational level was defined as low (illiterate and primary school), medium (secondary school and high school), and high (university). Also, the jobs were categorized into three levels (low, the jobs with no employment or demand for special education; medium, employed people without special education; and high, specialists).

### 2.3 Calculation of Framingham risk score

The 10‐year risk of CVD was calculated by FRS in the present study. Age, gender (man/woman), current smoking (yes/no), systolic blood pressure considering the current medication, high‐density lipoprotein‐cholesterol (mg/dl), and diabetes were included to assess FRS for the participants. The FRS is classified as follows: low risk: < 10%, intermediate risk: 10–19%, and high risk: ≥ 20% (D'Agostino Sr et al., 2008).

### 2.4 Calculation of Energy‐Adjusted Dietary Inflammatory Index

To measure the 31‐item E‐DII, a regional world database was employed to achieve an estimate of the mean and standard deviation (SD) for each item. The individual's food parameter consumption was deducted from the global standard mean and divided by its SD. The obtained value was converted to a centered proportion to minimize the effect of the “right skewing.” Then, the food factor inflammatory influence score was considered to achieve the participant's food parameter‐specific E‐DII score. Finally, these individual scores were summed to produce a total E‐DII score for each participant (Shivappa et al., 2014).

### 2.5 Statistical analysis

The data of the present study were recorded and analyzed in SPSS v.23. The qualitative and quantitative variables were reported as frequency (percent) and mean (standard deviation or standard error). The ANOVA and Chi‐square were used to compare the mean and frequency of variables among levels of FRS. Then, the unadjusted and adjusted association between E‐DII and FRS was investigated using multinomial regression. The level of significance was considered as *p* value <.05.

### 2.6 Ethics approval and consent to participate

The present study was approved by the Ethics Committee of Fasa University of Medical Sciences (Approval Code: IR.FUMS.REC.1401.097) and following the Helsinki Declaration. All participants were alerted about the aim of the research and fulfilled the written informed consent.

## 3 RESULTS

Among 10,030 participants eligible for this study, 4522 were men, and the mean age was 48.6 years (SD = 9.6). The mean E‐DII score of the studied population was −0.28 ± 2.07, ranging from −6.50 to 5.66. The median FRS was 2.80 (1.70, 6.30). Table 1 describes the characteristics of participants across the FRS groups. Participants were predominantly categorized as low risk (87.6%), and it was consistent in both genders. The proportion of women classified as low risk was significantly higher than for men (90.4% vs. 84.1%, *p*‐value <.001), and a higher percentage of men were classified as high risk in comparison with women. Men, widows, opium users, and smokers had higher FRS levels. Also, the participants with lower educational levels, socioeconomic status, and physical activity were more likely to have higher CVD risk.

**TABLE 1:** Comparison of characteristic features of the studied populations across the Framingham risk score classifications.

Variable	Groups	Total	Framingham risk score			
			Low risk (<10%)	Intermediate risk (10–19%)	High risk (≥20%)	*p‐*value^a^
		(*n* = 10,030)	(*n* = 8784)	(*n* = 973)	(*n* = 273)	
Age (years)		48.6 ± 9.6	46.9 ± 8.6	60.0 ± 5.8	64.0 ± 7.0	.000
Gender	Male	4522 (45.1%)	3805 (43.3%)	536 (55.1%)	181 (66.3%)	<.001
Body Mass Index (kg/m^2^)		25.6 ± 4.9	25.6 ± 4.9	26.2 ± 4.6	26.6 ± 4.5	<.001
Socioeconomic status (Assert index)		−0.01 ± 2.12	0.03 ± 2.13	−0.18 ± 2.01	−0.52 ± 1.77	<.001
Physical activity (MET)		41.5 ± 11.3	41.7 ± 11.3	40.1 ± 10.9	39.2 ± 12.2	<.001
Marital status	Single	363 (3.6%)	356 (4.1%)	5 (0.5%)	2 (0.7%)	<.001
	Married	8929 (89.0%)	7824 (89.1%)	864 (88.8%)	241 (88.3%)	
	Widow	636 (6.3%)	507 (5.8%)	100 (10.3%)	29 (10.6%)	
	Divorced	102 (1.0%)	97 (1.1%)	4 (0.4%)	1 (0.4%)	
Education	No education	4602 (45.4%)	3690 (42.0%)	703 (72.3%)	209 (76.6%)	<.001
	Primary school	4605 (45.4%)	4305 (49.0%)	241 (24.8%)	59 (21.6%)	
	Secondary school	800 (7.9%)	766 (8.7%)	29 (3.0%)	5 (1.8%)	
	University	23 (0.2%)	23 (0.3%)	0 (0.0%)	0 (0.0%)	
Diabetes		1238 (12.3%)	763 (8.7%)	327 (33.6%)	148 (54.2%)	<.001
Cardiac disease		1091 (10.9%)	798 (9.1%)	224 (23.0%)	69 (25.3%)	<.001
Alcohol		473 (4.7%)	441 (5.0%)	21 (2.2%)	11 (4.0%)	<.001
Opium consumption		2089 (20.8%)	1828 (20.8%)	197 (20.2%)	64 (23.4%)	.513
Smoking		548 (5.5%)	390 (4.4%)	106 (10.9%)	52 (19.0%)	<.001
Cholesterol (mg/dl)		185.1 ± 39.2	183 ± 38	198 ± 45	202 ± 52	<.001
High‐density lipoprotein (mg/dl)		131.9 ± 82.5	130 ± 80	145 ± 88	153 ± 122	<.001
Systolic Blood Pressure (mmHg)		111.4 ± 18.4	108 ± 15	133 ± 19	111 ± 18	.000

^a^ The quantitative variables were reported as Mean ± Standard Deviation (SD). The qualitative variables were reported as frequency (percent).

Table 2 demonstrates the dietary intake of participants among the FRS categories. Compared to the low‐risk participants (FRS < 10%), high‐risk participants (FRS ≥20%) consumed lower amounts of proteins, fibers, total fat, saturated fatty acid, monounsaturated fatty acids (MUFA), polyunsaturated fatty acids (PUFA), vitamin A, vitamin C, vitamin D, vitamin E, vitamin B12, vitamin B6, thiamin, selenium, zinc, magnesium, beta‐carotene, refined grains, onion, pepper, legumes, nuts, and white meat. However, high‐risk participants had a higher intake of high‐fat dairy in comparison with low‐risk participants. The difference in fruits, caffeine, and olive oil consumption did not show statistical significance across the FRS categories.

**TABLE 2:** Food intake of participants according to the Framingham risk score categories. (*n* = 10,030).

Food parameters	Framingham risk score			*p*‐value
	Low risk (<10%)	Intermediate risk (10%–19%)	High risk (≥20%)	
Nutrients				
Protein (g/d)	93.85 ± 37.93	85.04 ± 32.03	85.78 ± 35.67	.000
Fiber (g/d)	32.86 ± 14.21	29.01 ± 11.31	28.32 ± 11.44	.000
Total fat (g/d)	95.33 ± 38.06	85.35 ± 30.87	90.34 ± 40.24	.000
Saturated fatty acid (g/d)	26.18 ± 11.40	23.66 ± 9.35	25.32 ± 12.70	.000
MUFA (g/d)	30.41 ± 12.53	27.26 ± 10.23	29.26 ± 14.11	.000
PUFA (g/d)	21.54 ± 9.03	19.50 ± 7.46	20.50 ± 9.59	.000
Vitamin A (RAE/d)	616.88 ± 489.31	528.65 ± 436.04	492.65 ± 307.41	.000
Vitamin C (mg/d)	140.97 ± 100.51	117.78 ± 87.89	108.99 ± 73.51	.000
Vitamin D (mcg/d)	1.04 ± 1.07	0.93 ± 0.85	0.94 ± 0.84	.004
Vitamin E (mg/d)	19.24 ± 10.60	17.11 ± 8.92	18.45 ± 11.65	.000
Vitamin B12 (mcg/d)	4.14 ± 4.35	3.71 ± 3.98	3.54 ± 2.66	.001
Vitamin B6 (mcg/d)	2.10 ± 0.82	1.87 ± 0.66	1.87 ± 0.76	.000
Thiamin (mg/d)	3.10 ± 1.22	2.89 ± 1.07	2.88 ± 1.17	.000
Selenium (mg/d)	130.96 ± 55.13	121.1 ± 49.77	120.91 ± 51.35	.000
Zinc (mg/d)	12.63 ± 6.10	11.25 ± 5.15	11.44 ± 6.00	.000
Magnesium (mg/d)	412.99 ± 175.40	368.42 ± 145.08	368.90 ± 166.57	.000
Beta‐carotene (mcg/d)	3517.05 ± 2453.95	2899.00 ± 1885.76	2824.29 ± 1767.71	.000
Caffeine (mg/d)	192.54 ± 194.59	182.11 ± 165.86	181.64 ± 163.84	.191
Onion (g/d)	79.60 ± 79.11	67.18 ± 61.49	75.06 ± 74.68	.000
Pepper (g/d)	10.24 ± 24.37	8.01 ± 24.83	7.10 ± 12.72	.003
Food groups				
Refined grains (g/d)	373.15 ± 208.07	321.31 ± 190.34	314.76 ± 200.31	.000
Fruits (g/d)	382.16 ± 313.57	385.82 ± 319.18	352.89 ± 293.73	.288
High‐fat dairy (g/d)	153.92 ± 130.66	161.62 ± 139.03	172.32 ± 142.64	.021
Olive oil (g/d)	0.12 ± 0.72	0.17 ± 1.01	0.17 ± 0.69	.081
Legumes (g/d)	48.18 ± 39.06	45.87 ± 39.09	43.04 ± 35.91	.026
Nuts (g/d)	3.92 ± 8.25	2.82 ± 6.05	2.46 ± 5.70	.000
White meat (g/d)	72.83 ± 52.41	68.04 ± 52.086	72.45 ± 56.92	.026

*Note*: Quantitative variables are presented as mean ± standard deviation (SD). The *p*‐value was assessed by ANOVA for comparison of food parameters intake between different categories of Framingham risk score. *p*‐value <.05 was considered as statically significant.

Abbreviations: MUFA, monounsaturated fatty acids; PUFA, polyunsaturated fatty acids; RAE, retinol activity equivalent.

Table 3 shows that E‐DII score had a significant positive association with higher FRS. The association between E‐DII and high FRS remained significantly positive after adjustment for marital status, education, BMI, physical activity, alcohol consumption, opium use, and socioeconomic status. The adjusted model showed that 1 score increase in E‐DII score was associated with 24% higher chance of being in high‐risk group of FRS. Also, it was observed that the association of E‐DII with FRS was stronger among women than men.

**TABLE 3:** The association (odds ratio, 95% confidence interval) of Energy‐Adjusted Dietary Inflammatory Index (E‐DII) (as a continuous variable) with Framingham risk score (FRS).

Model	FRS level	Total		Men		Women	
		OR (95% CI)	*p‐*value	OR (95% CI)	*p‐*value	OR (95% CI)	*p‐*value
Crude	Intermediate	1.13 (1.09, 1.17)	<.001	1.11 (1.06, 1.17)	<.001	1.16 (1.11, 1.21)	<.001
	High	1.22 (1.15, 1.29)	<.001	1.27 (1.16, 1.38)	<.001	1.22 (1.11, 1.35)	<.001
Adjusted	Intermediate	1.14 (1.09, 1.19)	<.001	1.01 (0.94, 1.08)	.802	1.17 (1.10, 1.24)	<.001
	High	1.24 (1.15, 1.35)	<.001	1.17 (1.05, 1.30)	.005	1.19 (1.05, 1.36)	.009

*Note*: Adjusted for marital status, education, body mass index, physical activity, alcohol, opium, and socioeconomic status.

## 4 DISCUSSION

Our findings indicated that higher E‐DII scores were associated with a higher risk of CVD. Also, this association was stronger in women. Therefore, controlling the pro‐inflammatory potential of diet would have a promising preventive effect against CVD. Additionally, our study found that low‐risk individuals had a higher intake of fiber, vitamins, legumes, and nuts.

Previous studies showed a positive association between E‐DII and CVD. A cross‐sectional study on 276 Ecuadorian women showed that participants with a higher E‐DII have significantly higher FRS scores. Compared with our study, that study was conducted in a much smaller sample of women without any significant cardiometabolic conditions such as diabetes and hypertension with a lower mean age (Wang et al., 2021). A recent prospective study in the United Kingdom on 198,265 participants showed that a higher E‐DII score is significantly linked with a higher incidence of CVD. In line with our findings, the association between E‐DII score ≥ 0 and CVD incidence was still consistent after subgrouping the population by the age of 60 (Ho et al., 2023). Results from a cross‐sectional study in China indicated a significantly positive association between E‐DII and CVD (Ni et al., 2023).

Inflammation is crucial to the development of CVD and atherosclerosis (Libby, 2021a). Cholesterol augmentation in the arterial wall and endothelial dysfunction trigger the inflammatory process. Increasing concentrations of adhesion molecules such as vascular cell adhesion molecule‐1 (VCAM‐1) elicit immune cell migration, such as monocytes and lymphocytes (Alfaddagh et al., 2020; Libby, 2021a). Migrated lymphocytes turn into resident macrophages, which form a Nod‐like receptor protein 3 (NLRP3) inflammasome. Inflammasome formation activates pro‐IL‐1β and pro‐IL18 to IL‐1β and IL‐18, which then aggravate the inflammatory process by activating inflammatory cells and cytokines such as IL‐6 and CRP (Alfaddagh et al., 2020; Poston, 2019). This inflammation process leads to development, instability, and finally, rupture of atherosclerosis, which is the basis of CVD. The inflammatory response is mediated and developed by genetics, diet, smoking, obesity, diabetes, aging, and lack of exercise (Alfaddagh et al., 2020; Stojanović et al., 2020). So, theoretically, people with a more pro‐inflammatory diet should have an increased CVD risk, which is addressed in recent studies (Li et al., 2020).

The inflammatory pathophysiology of atherosclerosis made scientists focus on targeting inflammation for CVD prevention. Administration of appropriate doses of statins is indicated for individuals with high‐risk FRS (Kapur & Musunuru, 2008). Statins prevent atherosclerotic plaque formation, improve endothelial cell function, and prevent coronary artery diseases by their anti‐inflammatory effects (Diamantis et al., 2017). Moreover, colchicine showed encouraging effects in decreasing CVD risk as an anti‐inflammatory drug (Bonaventura & Abbate, 2023; Chen et al., 2023). In parallel with statin and anti‐inflammatory drugs, lifestyle modification, namely dietary interventions, has a major role in preventing CVD (Arnett et al., 2019). Previous studies showed that both healthy dietary advice and dietary approaches to stop hypertension (DASH) had a significant effect on the coronary artery disease prognosis of individuals (Said et al., 2021). Most of the recommendations to obey DASH have been focused on controlling hypertension as an important risk factor for CVDs (Challa et al., 2022). In addition, studies revealed that the Mediterranean diet decreases the chance of CVDs and mortality (Becerra‐Tomás et al., 2020). Both DASH and the Mediterranean diet mitigate inflammation (Itsiopoulos et al., 2022; Soltani et al., 2018), which could explain their preventive effects on CVD. Although controlling the amount of fat (Delgado‐Lista et al., 2022), salt (O'Donnell et al., 2020), and oxidant (Senoner & Dichtl, 2019) have been addressed in nutrition recommendations for preventing CVDs, there is limited evidence about the role of restricting pro‐inflammatory nutrients and increasing anti‐inflammatory nutrients in preventing coronary artery diseases (Liu & Dudley Jr, 2020). Consequently, in line with previous studies, our findings indicated that lowering E‐DII by dietary interventions would be promising in future studies.

### 4.1 Strengths and limitations

Our study had several advantages. First, this study involved a large number of participants (more than 10,000 people) from a well‐designed and precise cohort study. Second, comprehensive data collection in the FACS let us analyze different variables in this study to adjust the influence of confounding and mediating factors. Third, the inflammatory potential of the diet was scored using the E‐DII, which is controls for energy intake predicting the inflammatory potential (Hébert et al., 2019).

Like any other study, this study had some limitation that needs to be discussed. First, this study was a cross‐sectional study that could not be deduced with any causal conclusion. Second, the actual CVD risk assessment needs a longitudinal study, so FRS was used in this study. Therefore, further longitudinal studies are needed.

## 5 CONCLUSIONS

In conclusion, the present study suggests a significant association between CVD risk and pro‐inflammatory diet, which was relatively higher in women. Considering the importance of the inflammatory mechanisms in the progression of coronary artery disease, controlling the inflammatory potential of diet is suggested as in dietary intervention in prevention strategies for CVDs.

## AUTHOR CONTRIBUTIONS

**Matin Sepehrinia:** Conceptualization (equal); writing – original draft (equal). **Hossein Pourmontaseri:** Formal analysis (equal); writing – original draft (equal). **Mohammad Mehdi Naghizadeh:** Formal analysis (equal); methodology (equal). **Farhad Vahid:** Resources (equal). **James R. Hebert:** Resources (equal). **Reza Homayounfar:** Project administration (equal); resources (equal); supervision (equal); writing – review and editing (equal). **Abdulhakim Alkamel:** Conceptualization (equal); project administration (equal); writing – review and editing (equal).

## FUNDING INFORMATION

This research did not receive any specific grant from funding agencies in the public, commercial, or not‐for‐profit sectors.

## CONFLICT OF INTEREST STATEMENT

The authors declare no conflict of interest. However, Dr. James R. Hebert discloses that he owns controlling interest in Connecting Health Innovations LLC (CHI), a company that has licensed the right to his invention of the dietary inflammatory index (DII^®^) from the University of South Carolina in order to develop computer and smart phone applications for patient conseling and dietary intervention in clinical settings. CHI owns exclusive right to the E‐DII^TM^.

## Data Availability

Data Availability Statement: The data presented in this study are available on reasonable request from the corresponding author. The data are not publicly available due to privacy and ethical restrictions.
